# *RELN* Mutations in Autism Spectrum Disorder

**DOI:** 10.3389/fncel.2016.00084

**Published:** 2016-03-31

**Authors:** Dawn B. Lammert, Brian W. Howell

**Affiliations:** Department of Neuroscience and Physiology, SUNY Upstate Medical SchoolSyracuse, NY, USA

**Keywords:** *RELN*, Dab1, autism, autism spectrum disorder, brain development, cerebellum, neocortex

## Abstract

*RELN* encodes a large, secreted glycoprotein integral to proper neuronal positioning during development and regulation of synaptic function postnatally. Rare, homozygous, null mutations lead to lissencephaly with cerebellar hypoplasia (LCH), accompanied by developmental delay and epilepsy. Until recently, little was known about the frequency or consequences of heterozygous mutations. Several lines of evidence from multiple studies now implicate heterozygous mutations in *RELN* in autism spectrum disorders (ASD). *RELN* maps to the AUTS1 locus on 7q22, and at this time over 40 distinct mutations have been identified that would alter the protein sequence, four of which are *de novo*. The *RELN* mutations that are most clearly consequential are those that are predicted to inactivate the signaling function of the encoded protein and those that fall in a highly conserved RXR motif found at the core of the 16 Reelin subrepeats. Despite the growing evidence of *RELN* dysfunction in ASD, it appears that these mutations in isolation are insufficient and that secondary genetic or environmental factors are likely required for a diagnosis.

Autism spectrum disorder (ASD) currently affects as many as 1 in 45 children in the United States (Zablotsky et al., 2015). ASD incorporates Asperger, autism, and pervasive developmental disorder-not otherwise specified (PDD-NOS), and is characterized by social, behavioral, and language deficits. A small percentage (<20%) of ASD known as “syndromic autism” is attributable to monogenetic diseases, the two most common being fragile X syndrome and tuberous sclerosis (Miles, 2011; Persico and Napolioni, 2013). Other monogenetic disorders that have a high frequency of ASD but are less prevalent in the general population include Prader-Willi/Angelman, 15q microduplication, Rett, Smith-Lemli-Opitz, and Timothy syndromes.

The remaining 80% of ASD cases are considered “non-syndromic autism” and are the focus of high throughput sequencing efforts. A better understanding of how candidate genes contribute to ASD at the molecular level is key to understanding how so many variants converge on a common phenotype. *RELN*, encoding a large secreted glycoprotein, expressed in the brain and critical for proper brain development and synapse function, is consistently cited as a candidate gene for ASD (Persico and Napolioni, 2013).

In 2001 the International Molecular Genetic Study of Autism Consortium (IMGSAC) described a region on chromosome 7q as the peak region of linkage and first autism susceptibility locus (AUTS1; IMGSAC, 1998). Subsequent linkage studies supported this finding (IMGSAC, 2001a,b; Lamb et al., 2005). Given the role of *RELN* in neurodevelopment and its location at chromosome 7q22, *RELN* quickly emerged as a candidate gene for autism and numerous studies (>15) have investigated the occurrence of ASD risk-associated single nucleotide polymorphism (SNPs) in *RELN* (DeSilva et al., 1997; Persico et al., 2001; Krebs et al., 2002; Zhang et al., 2002). These and other studies had mixed results, possibly due to varying study designs, ethnic populations, and mathematical interpretations. A recent meta-analysis considered three known SNPs in *RELN*, and concluded that one rs362691 was significantly associated with an increased risk of ASD (Wang et al., 2014a).

While SNP analysis supports heterozygous mutations in *RELN*, it cannot explain how they contribute to ASD. Many genes have been proposed as candidates for ASD on the basis of sequencing analysis, but like *RELN*, their pathological mechanism remains speculative. Instead of perseverating on a particular individual SNP, researchers are now considering candidate genes on a much broader scale. The sum of coding and non-coding variants from genome-wide screens coupled with network analyses, gene and protein expression, and epigenetic modifications provide evidence that helps understand functionally how a gene contributes to ASD (Neale and Sham, 2004). From these types of analyses emerges an approach for deciphering the role of *RELN* in ASD at the molecular level, beyond association.

*RELN* expression is both spatially and temporally consistent with ASD, which is thought to originate as a neurodevelopmental disorder that persists into postnatal life. Homozygous loss of *RELN* leads to severe neuronal dysplasia in several brain regions including the neocortex, hippocampus, and cerebellum. Patients homozygous null for *RELN* suffer from lissencephaly with cerebellar hypoplasia (LCH), a profoundly developmentally debilitating disease (Hong et al., 2000; Chang et al., 2007). Patients with LCH also suffer from epilepsy, but no autistic behavior has been reported in the patients or their parents.

*RELN* is first expressed by Cajal Retzius (CR) cells, and other less well defined marginal zone neurons, that act as pioneer neurons by regulating the positioning of projection neurons into discrete layers in the neocortex (D’Arcangelo et al., 1995; Hirotsune et al., 1995; Ogawa et al., 1995; Ikeda and Terashima, 1997; Meyer et al., 2004). CR eventually degenerate and a population of GABA-ergic interneurons expresses *RELN* postnatally (Pesold et al., 1998). In the developing cerebellum, *RELN* is first expressed by cells of the rhombic lip that migrate to populate the external granule layer and regulate the position of Purkinje neurons (D’Arcangelo et al., 1995; Miyata et al., 1997). Postnatally, cerebellar granule cell neurons (GCNs) now positioned in the internal granule layer continue to secrete Reelin, although its postnatal role is not clear (Sinagra et al., 2008). The brain size and architecture are relatively normal in the heterozygous reeler mouse (HRM); however, male HRM, thought to model ASD, have decreased numbers of Purkinje cells (Hadj-Sahraoui et al., 1996; Biamonte et al., 2009).

Traditionally, the cerebellum is considered responsible for fine-tuning movement, but its role in cognitive and emotional functions is now appreciated (Buckner, 2013). Acute adult injury results in the classical cerebellar signs early, such as asynergy, followed by subtle, often overlooked cognitive and communication impairments. In contrast, damage to the cerebellum during development leads to cognitive and communication defects. Interestingly, these early injuries have also been associated with the occurrence of ASD, which highlights a role for the cerebellum in its etiology (Becker and Stoodley, 2013; Wang et al., 2014b).

One of the most consistent anatomic findings in ASD is a decrease in cerebellar Purkinje cells and decreased volume of the vermis (Fatemi et al., 2012; D’Mello et al., 2015; Hampson and Blatt, 2015). In the most recent stereologic study, Skefos et al. (2014) found that Purkinje cells were decreased in ASD individuals. Compared to previous studies, this group included patients with cognitive delay and epilepsy, showing that this defect is widespread across ASD. The role of Purkinje cells and their ability to drive ASD behaviors as seen in tuberous sclerosis, has been recently demonstrated in a mouse model (Tsai et al., 2012a). They used a conditional knock-out of TSC1 in Purkinje cells to show that dysfunction in these cells was sufficient to decrease interest in novel mouse social interaction, increase grooming, and increase ultrasonic vocalizations—behaviors consistent with other ASD mouse models (Silverman et al., 2010).

Not only is *RELN* expression consistent with ASD, but the Reelin signaling pathways intersect prominent ASD protein networks. To understand how so many disparate genes can converge on a similar phenotype, grouping candidate genes into networks has helped to uncover cellular processes that might be driving ASD. Network analysis continually implicates synaptic function and dysregulated protein translation, particularly at the synapse (Gilman et al., 2011; Sanders et al., 2012; Ebert and Greenberg, 2013; De Rubeis et al., 2014).

Canonical Reelin signaling is initiated by Reelin binding its receptors very-low-density-lipoprotein receptor (VLDLR) and apolipoprotein E receptor 2 (ApoER2; LRP8; D’Arcangelo et al., 1999; Hiesberger et al., 1999; Trommsdorff et al., 1999). Disabled-1 (Dab1) is recruited to the receptors, which then activates Src family kinases and leads to reciprocal activation through tyrosine phosphorylation of Dab1 (Howell et al., 1997, 1999; Rice et al., 1998; Arnaud et al., 2003; Bock and Herz, 2003). This signaling initiates a number of signaling cascades, which have been extensively reviewed (Tissir and Goffinet, 2003; D’Arcangelo, 2014; Sekine et al., 2014). Dab1 and VLDLR have been suggested to be associated with ASD risk, but overall there is little evidence directly implicating Dab1, VLDLR, ApoER2, SRC, or FYN in ASD (Fatemi et al., 2005; Iwata et al., 2012; Li et al., 2013; Shen et al., 2015).

Particularly relevant to ASD network analyses are recent studies of the integral role of Reelin as a modulator of the postnatal synapse and the ability of Reelin to enhance LTP in the hippocampus (Weeber et al., 2002; Beffert et al., 2005; Chen et al., 2005). Secretion of Reelin, however, is constitutive and independent of synaptic activity (Lacor et al., 2000). Canonical Reelin-ApoER2/VLDLR-Dab1 signaling leads to phosphorylation of the NMDA receptor (NMDAR), increased calcium flux with glutamate stimulation, as well as altered intermembrane mobility of NR2B and NR2A subunit-containing receptors (Beffert et al., 2005; Chen et al., 2005; Groc et al., 2007; Campo et al., 2009; Ventruti et al., 2011). Reelin is also capable of modulating presynaptic neurotransmitter release by regulating the VAMP7 and SNAP-25 interaction (Bal et al., 2013).

Structurally, ApoER2, NMDAR, PTEN, and PSD-95 form a complex at the post-synaptic density in a Reelin-dependent manner (Ventruti et al., 2011; Figure 1). Neurexins and neuroligins, pre- and post-synaptic cell adhesion molecules respectively, organize the synapse, and each has been implicated in ASD (Dean and Dresbach, 2006). Neuroligins interact with PSD-95, which in turn is anchored to the cytoskeleton through SHANK proteins (Ebert and Greenberg, 2013). SHANK3 mutations often lead to Phelan-McDermid syndrome, which frequently presents with ASD (Grabrucker et al., 2011). PSD-95 expression is also regulated by fragile X mental retardation protein (FMRP), the protein implicated in fragile X syndrome (Tsai et al., 2012b). Hypermethylation of a trinucleotide expansion leads to decreased expression of FMRP and subsequent augmented synaptic mRNA translation.

**Figure 1:**
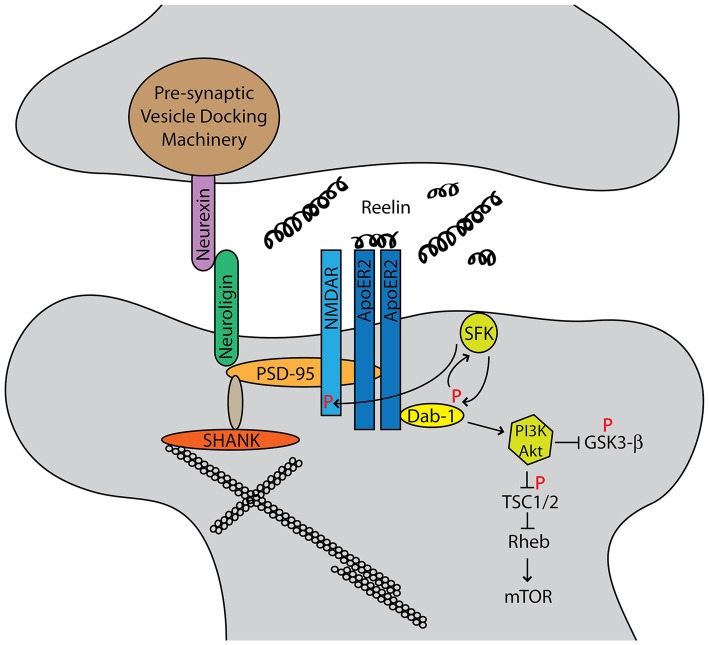
**Autism spectrum disorders (ASD) candidate genes cluster into functional networks, and the two most prominent are synapse structure/function and protein translational control.** The Reelin-signaling pathway intersects both of these networks. Reelin binds its receptors ApoER2 and very-low-density-lipoprotein receptor (VLDLR). The adapter protein Dab1 binds the cytoplasmic NPXY motif of the receptors and is phosphorylated by Src family kinases. This reciprocally activates Src, which leads to phosphorylation of the NMDA receptor (NMDAR) as well as downstream AKT/PI3K signaling that intersects the mTOR pathway.

Reelin also directly intersects protein translation control, the second major candidate gene network and hallmark of Fragile X and tuberous sclerosis syndromes. Tuberous sclerosis is caused by mutations in either TSC1 or TSC2 genes, which leads to hyperactivation of mTORC1 and subsequent increases in protein translation (Crino, 2011). Canonical Reelin signaling activates Akt, which phosphorylates TSC1/2 and leads to dendrite growth and branching (Jossin and Goffinet, 2007). Recently, Reelin and Dab1 protein expression were shown to be increased in TSC2 conditional knock-out mice as well as human cortical tubers (Moon et al., 2015). Here, activation of mTOR signaling may impair Cul5-mediated Dab1 degradation. Although Reelin signaling through mTOR is still incompletely understood, it is clear that it plays a significant role.

Further evidence for *RELN* involvement in ASD is the observation of decreased expression of *RELN* transcript and encoded protein in ASD patients. Decreased Reelin was detected in the cerebellum of ASD subjects as compared to controls (Fatemi et al., 2001, 2005) and in the superior frontal cortex (Fatemi et al., 2005). *RELN* mRNA in these areas was decreased, as was the *dab1* transcript. *VLDLR* mRNA levels were increased.

Part of the elusiveness of ASD etiology is the likelihood of gene-environment interactions. Maternal stressors during gestation have been shown to alter *RELN* expression through promoter methylation (Giovanoli et al., 2014). MeCP2, the gene implicated in Rett and MeCP2 duplication syndromes, which share features of ASD, shows increased binding to the *RELN* promoter in human ASD cerebella (Zhubi et al., 2014). This binding corresponds with decreased *RELN* mRNA expression, consistent with the aforementioned reduced *RELN* expression in ASD tissue samples.

With the advent of more efficient and affordable sequencing technologies, whole-exome sequencing (WES) has become a new, popular approach for identifying candidate genes. WES identifies probable disease-contributing mutations that disrupt protein function. The average rate of mutation for the human genome is 1.2 × 10^−8^ per nucleotide. Over the entire genome, Kong et al. (2012) detected 63.2 *de novo* mutations per trio studied, but only 2% of the human genome is actually coding sequence (Kong et al., 2012). Therefore, in agreement with these findings, each exome has approximately only a single *de novo* protein changing allele (Gratten et al., 2013). Focusing then on only detected *de novo* events is a way to streamline candidate gene discovery.

Initial expectations were that individuals with yet unsolved complex disorders would have increased indels, CNVs, and frameshift, nonsense, and missense mutations compared to controls. While findings support that nonsense mutations may be more frequent in ASD than controls, the general finding is that there is not a dramatic overall increase in *de novo* mutation rates in ASD (Neale et al., 2012; Sanders et al., 2012; Samocha et al., 2014). Furthermore, *de novo* mutations do not make up a large enough proportion of cases to explain the elusive genetics of ASD, and likely represent less than 5% of the overall ASD risk (Neale et al., 2012).

Currently more than two *de novo* mutations in a gene support its candidacy, although this threshold will increase with increasing patients to control for multiple testing. *RELN* currently has four unique documented *de novo* ASD-associated mutations, three of which are likely pathological (Neale et al., 2012; De Rubeis et al., 2014; Iossifov et al., 2014; Yuen et al., 2015). Furthermore, *RELN* was 1 of 22 genes with a false discovery rate of < 0.05 in a study of nearly 4000 ASD patients (De Rubeis et al., 2014). *De novo* mutations, while directly explaining very few cases, are likely to contribute, at least in part, to disease in the proband in whom they were discovered. Given the repeated implication of particular gene signaling networks in ASD, understanding how a single *de novo* mutation influences this system at the molecular level will help explain a much larger number of ASD cases (Gratten et al., 2013).

Large and small scale WES studies of ASD individuals consistently identify missense and nonsense mutations in *RELN*, leading researchers to emphasize its importance in ASD (De Rubeis et al., 2014). There are currently over 40 unique *RELN* variants identified in ASD probands that are absent in controls (Bonora et al., 2003; Neale et al., 2012; Koshimizu et al., 2013; De Rubeis et al., 2014; Iossifov et al., 2014; Yuen et al., 2015; Zhang et al., 2015; Figure 2). These mutations have not been functionally characterized; however, strong predictions regarding their consequences can be deduced based on Reelin structure and function.

**Figure 2:**
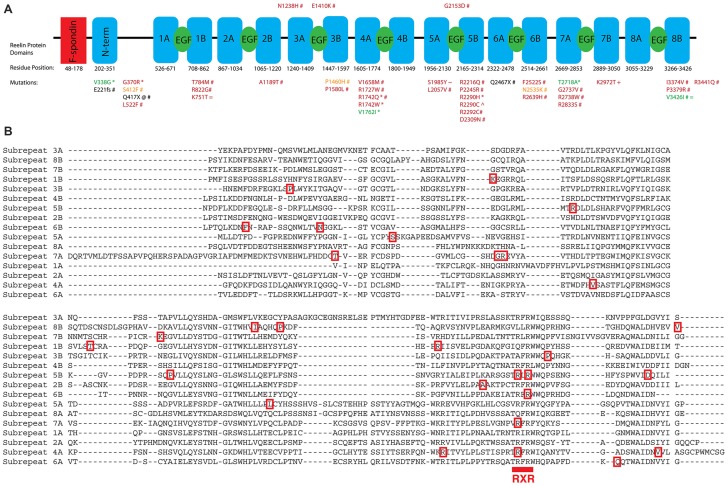
**The structure of Reelin is diagrammed with conserved domain boundaries mapped (NCBI Conserved Domain Database). (A)** Missense, nonsense, and frameshift mutations that are absent in controls and thus more likely to contribute to ASD are indicated (*Bonora et al., 2003; ^@^Neale et al., 2012; ^+^Koshimizu et al., 2013; ^#^De Rubeis et al., 2014; ^∧^Iossifov et al., 2014; ^~^Yuen et al., 2015; ^=^Zhang et al., 2015). Specifically, mutations identified by Bonora et al. (2003) and De Rubeis et al. (2014) as occurring in isolated controls from case-control studies, even if they overlap with mutations identified in other studies, are not pictured. This process did not remove any RXR mutations identified in ASD probands; however, three RXR mutations were identified in controls: R1198H, R2104H, and R2292H. Missense mutations are colored based on PolyPhen2 predictions (green, benign; orange, possibly damaging; red, probably damaging). Nonsense and frameshift mutations are indicated in black. **(B)** Clustal Omega alignment of the sub-repeat domains is annotated with the corresponding missense mutations from **(A)**. The RXR consensus sequence is indicated below the aligned repeat sequence. Note: corrections to annotations for missense mutations from supplementary file S4 of De Rubeis et al. (2014) are as follows: R3439Q to R3441Q, G254V to G2737V, R156H to R2639H.

Reelin, a large 410 kDa protein, comprises eight Reelin repeat domains (D’Arcangelo et al., 1995; Nogi et al., 2006; Panteri et al., 2006; Yasui et al., 2007). Each Reelin repeat domain is composed of two subrepeat domains (A and B) linked by an EGF-like domain that share highly conserved sequences and are structurally similar. Reelin binds its receptors ApoER2 and VLDLR through two lysine residues on subrepeat 6A (Yasui et al., 2010).

The first prediction from structure-function analysis is that any nonsense mutation that truncates Reelin before the receptor-binding domain will be loss-of-function. In this instance, the transcript would either be degraded by nonsense-mediated decay or it would produce a protein product unable to initiate canonical signaling. Two mutations with this characteristic have been identified—a *de novo* mutation Q417X and a frameshift mutation that disrupts Reelin after E221 (De Rubeis et al., 2014). Both of these mutations occur before the receptor binding residues K2359 and K2466 (mouse equivalents K2360, K2467; Yasui et al., 2010). Whether these mutations could also contribute to a possible gain-of-function, perhaps through a non-canonical Reelin pathway is unclear, since the receptor-ligand domain is unknown (Lee et al., 2014).

Alternatively, one may predict that mutations may interfere with conserved domains, altering Reelin function in a way that contributes to the ASD phenotype. Aligning the subrepeat sequences of Reelin (Clustal Omega) and plotting the mutations identified in ASD genetic studies, we have found that Reelin is enriched in mutations that lie within an RXR consensus sequence that occurs once in each subrepeat (Bonora et al., 2003; De Rubeis et al., 2014; Iossifov et al., 2014; Figure 2). Of the identified variants, seven unique mutations fall within the RXR consensus sequence—a much larger percentage than would be expected by chance (R1742W, R1742Q, R2290C, R2290H, R2292C, R2639H, R2833S). R2290C, a mutation falling within the RXR consensus sequence, was discovered as a *de novo* variant originating on the paternal chromosome (Iossifov et al., 2014). This RXR consensus region is highly conserved across evolution, suggesting a particular functional relevance for this region that is linked to ASD pathogenesis.

Each subrepeat is composed of an 11-stranded beta-jelly roll fold, and the RXR consensus sequence is found at the beginning of the 10th beta sheet (Nogi et al., 2006). Arginine is important structurally for hydrogen bonding with the protein backbone (Borders et al., 1994). Disruption at this position could compromise protein folding, exposing the hydrophilic pore and enabling novel interactions. Alternatively, considering that Reelin may serve as an extracellular matrix (ECM) molecule, these closely spaced arginines and their neighboring tryptophan residues may be important for glycosaminoglycan binding (Panteri et al., 2006).

Clustering of mutations within this RXR consensus sequence argues against random mutations leading to complete loss-of-function. Presumably many mutations throughout the 3460 amino acids of Reelin could disrupt function. Therefore, a mutational hotspot might suggest a particular mode of loss-of- or gain-of-function, the details of which will need to be determined experimentally.

Animal models of *RELN* mutations may ultimately be necessary to parse out the link between *RELN* and ASD. Thus far, simple loss-of-function alleles have not provided overwhelming evidence that heterozygous *RELN* mutations in the mouse produce overt or consistent behavioral phenotypes reminiscent of ASD (Moy and Nadler, 2008). Similarly, the human genetics of *RELN* mutations suggests that a second hit, either environmental or genetic, may be necessary for ASD. Parents of patients with LCH are heterozygous for *RELN* loss-of-function alleles but do not have ASD (Hong et al., 2000; Chang et al., 2007). Approximately half of the ASD-associated mutations identified in *RELN*, including truncating and RXR mutations, are inherited from normal parents. In addition, following the same method of characterizing mutations in controls from ASD studies, here too there are examples of a nonsense mutation truncating Reelin before the receptor binding domain (Q849X) and RXR consensus mutations (R1198H, R2104H, and R2292H; Bonora et al., 2003; De Rubeis et al., 2014).

Since *RELN* is particularly susceptible to environment-driven epigenetic changes, one can hypothesize that perhaps a single mutation, which decreases its expression, combined with environmental down-regulation of Reelin production, could drive Reelin protein levels below a critical threshold in the brain. Or, perhaps another modifying gene allele in trans provides this added susceptibility. One likely contributing factor is sex. *RELN* mutations occur in approximately four times as many male as female probands. And indeed, testosterone and estrogen have differing effects on *RELN* expression and HRM phenotypes (Hadj-Sahraoui et al., 1996; Absil et al., 2003; Biamonte et al., 2009; Macri et al., 2010).

Adding to the excitement and promise of deciphering the role of *RELN* in ASD is evidence that Reelin supplementation or increased production could potentially reverse behavioral consequences of decreased Reelin signaling (Rogers et al., 2011, 2013; Hethorn et al., 2015). As mutations in *RELN* continue to be identified in genetic studies and the molecular mechanisms of these mutations are elucidated, we will better understand the role of Reelin in neuronal signaling, development, and ASD.

In the same way that science stresses transitioning from the bench to the bedside, we must also start to move gene candidates from the computer to the bench. *RELN* is now well positioned to make such a transition in ASD research. This approach will not be without its challenges, since it is predicted that for *RELN* and many of the other candidate genes, a single mutation may not be sufficient to cause overt ASD phenotypes, and gene-gene or gene-environment interactions will need to be considered.

## Author Contributions

DBL wrote the manuscript and BWH suggested the topics to be covered and edited its content.

## Funding

This work was supported by R01 NS073662 (BWH) and F31 NS086731 (DBL). National Institute of Neurological Disorders and Stroke/National Institutes of Health.

## Conflict of Interest Statement

The authors declare that the research was conducted in the absence of any commercial or financial relationships that could be construed as a potential conflict of interest.
